# Examination of the Metallothionein Gene Family in Greater Duckweed *Spirodela polyrhiza*

**DOI:** 10.3390/plants12010125

**Published:** 2022-12-27

**Authors:** Orathai Pakdee, Shomo Tshering, Prayad Pokethitiyook, Metha Meetam

**Affiliations:** 1 Department of Biology, Faculty of Science, Mahidol University, Bangkok 10400, Thailand; 2 Center of Excellence on Environmental Health and Toxicology (EHT), OPS, MHESI, Bangkok 10400, Thailand

**Keywords:** duckweed, heavy metal, metallothionein

## Abstract

Duckweeds are aquatic plants that proliferate rapidly in a wide range of freshwaters, and they are regarded as a potential source of sustainable biomass for various applications and the cost-effective bioremediation of heavy metal pollutants. To understand the cellular and molecular basis that underlies the high metal tolerance and accumulation capacity of duckweeds, we examined the forms and transcript profiles of the metallothionein (MT) gene family in the model duckweed *Spirodela polyrhiza*, whose genome has been completely sequenced. Four *S. polyrhiza* MT-like genes were identified and annotated as *SpMT2a*, *SpMT2b*, *SpMT3*, and *SpMT4*. All except *SpMT2b* showed high sequence homology including the conserved cysteine residues with the previously described MTs from flowering plants. The *S. polyrhiza* genome appears to lack the root-specific Type 1 MT. The transcripts of *SpMT2a*, *SpMT2b*, and *SpMT3* could be detected in the vegetative whole-plant tissues. The transcript abundance of *SpMT2a* was upregulated several-fold in response to cadmium stress, and the heterologous expression of *SpMT2a* conferred copper and cadmium tolerance to the metal-sensitive ∆*cup1* strain of *Saccharomyces cerevisiae*. Based on these results, we proposed that *SpMT2a* may play an important role in the metal detoxification mechanism of duckweed.

## 1. Introduction

Members of the metallothionein (MT) gene family encode low-molecular-weight, cysteine (Cys)-rich proteins and are believed to play various important roles in the homeostasis of metals and reactive oxygen species (ROS) in eukaryotic organisms and some bacteria. The Cys residues of MT proteins have been shown to coordinate metal ions in various configurations depending on the metal load, suggesting that MTs generally function as intracellular metal chelators for metal detoxification, distribution, and/or storage [1]. The Cys residues can also participate in the scavenging of ROS under oxidative stress [2]. Another unique characteristic of the MT proteins is the arrangement of the Cys residues that are highly conserved among the related MT homologs but differ between the MT gene lineages. In angiosperms, four lineages of MT genes, referred to as Types, have been described based on the conserved Cys arrangements of the gene products [3]. Studies on various flowering plants have shown that all four MT Types are present in the plants, but they differ in their expression patterns. For instance, Type 1 MTs are usually expressed in root tissues and Type 4 MTs are primarily expressed in seeds. The conservation in the amino acid sequences and expression patterns among the MT gene lineages clearly suggest that they inherit specific and indispensable functions [4]. However, the essential functions of each MT lineage in various organisms remain largely elusive.

Duckweeds are small aquatic flowering plants of the family *Lemnaceae*, with 36 plant species encompassing the following five genera: *Spirodela*, *Landoltia*, *Lemna*, *Wolffiella*, and *Wolffia* [5]. Due to their high proliferation rate and nutrient richness, duckweeds have recently attracted attention as sustainable sources of livestock feed, human nutrition, and renewable biomass for the biofuel production and green industries [6]. Another advantage of duckweeds over traditional crops is their ability to grow in eutrophic water and simultaneously remove undesirable pollutants [7]. Heavy metal contamination in water due to anthropogenic activities is a grave concern in the present century. The accumulation of heavy metals in water can have adverse effects on the health of all the aquatic organisms, as well as humans [8]. Some duckweeds are considerably metal-tolerant plant species, and many studies have indeed shown the high efficiency and cost-effectiveness of duckweeds in heavy metal removal applications [9]. Despite the great potential of duckweeds in heavy metal bioremediation, little is known about the cellular and molecular mechanisms that enable to duckweeds to efficiently take up, accumulate, and detoxify the heavy metals from the environment (for recent studies on the metal homeostasis network in duckweeds, see [10,11]).

In this study, the forms and potential functions of MTs were investigated in the greater duckweed *Spirodela polyrhiza*. The expression and transcriptional responses of the different *S. polyrhiza* MT-like genes to metal stress were examined. To test whether the protein products from the putative *S. polyrhiza* MT genes can serve as intracellular metal chelators, the effect of the heterologous expression of the *S. polyrhiza* MT homologs in the yeast *Saccharomyces cerevisiae* mutant ∆*cup1*, which lacked the major endogenous MT gene, was investigated.

## 2. Results

### 2.1. Identification and Annotation of Putative S. polyrhiza MT-like Genes

**Figure 1:**
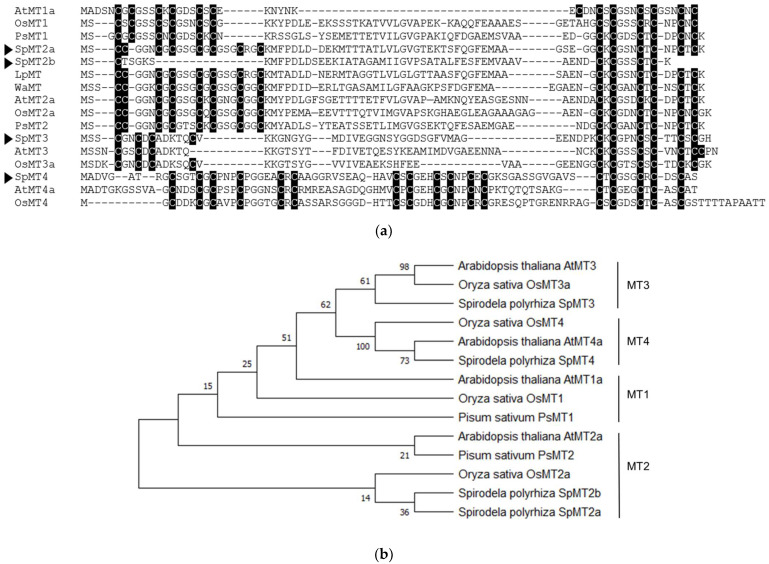
Identification of MT-like genes from *S. polyrhiza*: (**a**) alignment of predicted amino acid sequences of *S. polyrhiza* MT homologs against representative vascular-plant MTs; (**b**) a neighbor-joining tree based on the sequence alignment, with bootstrap values from 1000 iterations. Cysteine residues are highlighted in black. Arrows indicate the *S. polyrhiza* MT homologs. The accession numbers of the sequences used in the analysis are: AtMT1a (837273), OsMT1 (U43529), PsMT1 (BAD18382), LpMT (JZ977403.1), WaMT (JK990501.1), AtMT2a (820098), OsMT2a (P94029), PsMT2a (BAD18383), AtMT3 (820772), OsMT3a (A1YTM8), AtMT4a (818800), and OsMT4 (Q109B0).

The MT-like genes in the genome of *S. polyrhiza* were first identified using a keyword search in the Phytozome database. The search resulted in four predicted transcripts: Spipo6G0071500, Spipo0G0175800, Spipo0G0112500, and Spipo14G0028700. The searches for additional *S. polyrhiza* MT-like genes were performed using the known sequences of the MT gene family from *Oryza sativa* and *Arabidopsis thaliana*, as well as some previously described MT sequences from representative monocot species, against the genomes of *S. polyrhiza* in the GenBank (NCBI) database, but no additional MT-like genes were identified. To annotate the *S. polyrhiza* MT-like genes, their predicted amino acid sequences were aligned against the known MTs from representative plant species (Figure 1a) and a neighbor-joining tree was constructed (Figure 1b). Based on these results, the *S. polyrhiza* MT-like genes were annotated (Table 1). Interestingly, no homolog of Type 1 MTs could be identified, suggesting that the *S. polyrhiza* genome lacks one of the typical MT lineages that are found in the genomes of flowering plants. Two Type 2 MT homologs were found in *S. polyrhiza*. The predicted amino acid sequence of Spipo0G0112500, annotated as *SpMT2a*, shared all of the fourteen Cys residues that are found in other flowering plants such as *O. sativa* and *A. thaliana*. In contrast, the predicted amino acid sequence of Spipo6G0071500, annotated as *SpMT2b*, only shared five of the fourteen Cys residues. As with SpMT2a, other Type 2 MT homologs from other duckweed species such as *Landolita punctata* and *Wolffia australiana* were also found to share all of the fourteen Cys residues. This suggests that *SpMT2b* does not encode a bona fide MT. Spipo14G0028700 and Spipo0G0175800 were found to encode single-member Type 3 and Type 4 MTs, respectively. The predicted amino acid sequences of *SpMT3* and *SpMT4* shared all of the critical Cys residues conserved among the flowering plant species. In addition, SpMT4 shared the two conserved histidine residues that are typically found in the middle Cys-rich domain of Type 4 MTs and which are potentially involved in metal coordination [12]. It should be noted that SpMT4 contains a stretch of nine non-Cys amino acids prior to the N-terminal Cys-rich domain. This extended N-terminal sequence is commonly found in the Type 4 MTs from dicot plants, but it is absent in OsMT4 and other Type 4 MTs from several monocots including wheat, maize, and barley [12]. This prompted us to investigate whether the extended N-terminal sequence found in SpMT4 is unique to the duckweed MT. A TBLASTN search using the SpMT4 amino acid sequence against the NCBI genome databases showed that SpMT4 was most closely related to a Type 4 MT from *Spirodela intermedia*, followed by Type 4 MTs from two other monocot plants—*Xerophyta humilis* and *Elaeis guineensis*—all of which contain an extended N-terminal sequence prior to the Cys-rich domain (data not shown). Therefore, SpMT4 is not the only monocot Type 4 MT that harbors the extended N-terminal sequence.

**Figure 2:**
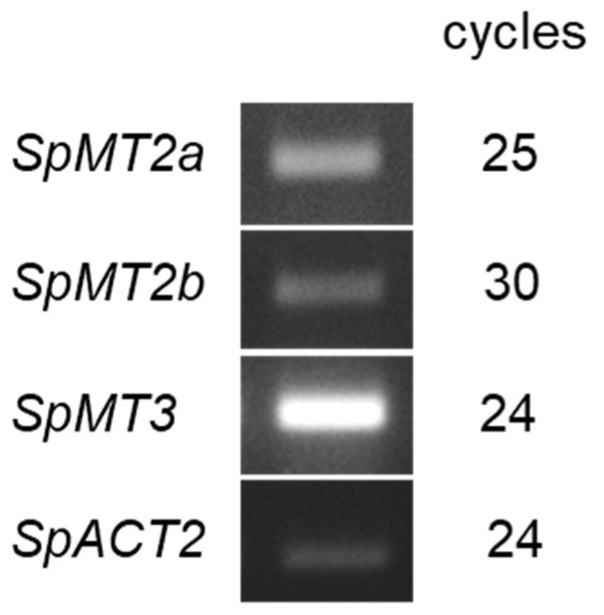
Semi-quantitative RT-PCR analysis of transcript abundance in the whole plant tissues of *S*. *polyrhiza* cultured in normal Hoagland solution. *SpACT2* was included as a standard control. The number of cycles used in the PCR reactions are indicated.

To confirm that the MT-like *S. polyrhiza* genes were expressed at the transcript level, the RT-PCR analysis was carried out using a total cDNA extract from the whole of *S. polyrhiza* plant. The transcripts of all three *S. polyrhiza* MT genes, but not of *SpMT4*, could be detected in the whole plant tissues, confirming that they are functionally transcribed (Figure 2). It is possible that the transcript of *SpMT4* was not detected in the whole plant tissues because the expression of Type 4 MTs is typically restricted to seeds [3]. However, the potential expression of *SpMT4* in the seeds of *S. polyrhiza* could not be investigated in this study due to the unavailability of the duckweed seeds at the time of the experiment. 

### 2.2. Transcriptional Response to Cadmium and Copper Stresses

**Figure 3:**
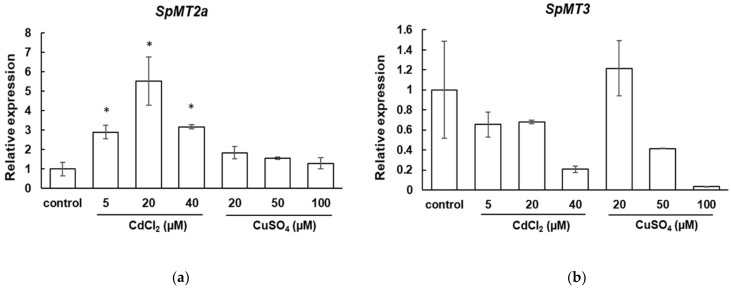
Transcript abundance of *S. polyrhiza* MT genes in whole plant tissues under metal stress: (**a**) *SpMT2a*. (**b**) *SpMT3*. The *S. polyrhiza* plants were cultured in Hoagland solution supplemented with the indicated concentrations of CdCl_2_ or CuSO_4_ for 24 h. The relative transcript abundance was quantified and normalized to that of *SpACT2*. The relative expression of the transcript abundance was adjusted to the control level. The asterisks indicate values that were significantly higher than the control condition and exceeded a fold change of two (one-tailed *t*-test, *p* < 0.05). The error bars represent SE (n = 3).

The expression of plant *MT* genes is often induced by heavy metals, suggesting their role in heavy metal detoxification and tolerance. To investigate whether any of the *S. polyrhiza* MT genes might be functionally involved in the metal detoxification mechanism, the transcriptional responses of *SpMT2a* and *SpMT3* to heavy metal stress were examined. The *S. polyrhiza* plants were cultured for 24 h in medium containing various concentrations of CuSO_4_ and CdCl_2_ (Figure 3). The transcript abundance of *SpMT2b* was not investigated because it does not appear to encode a functional MT protein, whereas *SpMT4* was not expressed in the vegetative tissues, as demonstrated previously. The quantitative RT-PCR analysis showed that the transcript abundance of the *SpMT2a* genes was significantly up-regulated, more than two-fold (*p* < 0.05), by the CdCl_2_ treatments. The CuSO_4_ treatment slightly induced the *SpMT2a* expression, but not to a level that was statistically significant compared to the control. In contrast, the expression of *SpMT3* was not induced by the CuSO_4_ or CdCl_2_ treatments, and interestingly, it was partially down-regulated in response to high concentrations of CuSO_4_ or CdCl_2_. These results suggest that *SpMT2a* may play a role in the ability of *S. polyrhiza* to tolerate cadmium.

### 2.3. Copper and Cadmium Tolerance Conferred by the Heterologous Expression of SpMT2b in S. cerevisiae

**Figure 4:**
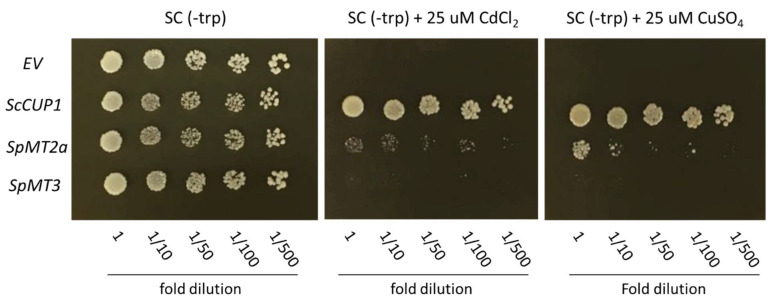
Metal sensitivity of the *S. cerevisiae* ∆*cup1* strain expressing the *SpMT2a* and *SpMT3* genes in comparison to the controls: empty vector (EV) or *S. cerevisiae CUP1* (Sc*CUP1*). Serially diluted yeast cultures were spotted on normal SC(-trp) agar or the medium supplemented with 25 µM CdCl_2_ or 25 µM CuSO_4_ for 3 days.

We previously showed that the *S. polyrhiza* MT genes, except *SpMT2b*, shared with other plant MT homologs the conserved metal-binding motifs which may participate in metal chelation and contribute to the function of MT proteins in duckweed’s metal homeostasis mechanism. To further test whether the protein products encoded by some of the *S. polyrhiza* MT genes could indeed function as metal chelators in vivo, we constitutively expressed *SpMT2a* and *SpMT3* in the *S. cerevisiae* copper-sensitive ∆*cup1* mutant, which lacked one of its two MT genes. The dilution spot assay showed that growth of the ∆*cup1* strain transformed with the empty p424-GPD vector was completely inhibited in medium supplemented with either 25 µM CuSO_4_ or 25 µM CdCl_2_ (Figure 4). For a positive control, the ∆*cup1* strain was transformed with the vector p424-GPD which harbored the yeast *CUP1* gene. The *CUP1* complement restored the copper and cadmium tolerance of the ∆*cup1* mutant. The heterologous expression of *SpMT2a* in the ∆*cup1* mutant could also confer growth tolerance to the cadmium and copper stress, although to a lower extent compared to the *CUP1* gene, indicating that the protein product of *SpMT2a* could function as a metal chelator *in vivo*. In contrast, the ∆*cup1* mutant that expressed *SpMT3* did not appear to grow under the metal stress under these conditions, although it should be noted that the inability of the *SpMT3* heterologous expression to impart metal tolerance in the yeast ∆*cup1* mutant could be attributed to several unforeseen reasons such as the failure of the duckweed protein to efficiently express inside the yeast cells.

## 3. Discussion

Heavy metal contamination in aquatic environments is a global concern. The experimental application of various duckweeds in the phytoremediation of polluted water has been met with considerable success and improved the water parameters, including heavy metals reduction [7,13,14]. For instance, it has been reported that *Lemna gibba* was able to remove >90% of Ni, Pb, and Cd at industry-relevant metal concentrations [15]. The actual removal efficiency may depend on many factors, including duckweed species, metal species, initial metal concentration, and other water parameters. The duckweed byproduct can also be utilized in several applications including as biomass for biofuel and as biomaterial and in plant factories for the production of high-value bioproducts [15,16]. In general, duckweeds are considered promising candidates for the metal phytoremediation due to their high capacity for metal tolerance, accumulation, and bioconcentration factors [17,18,19]. Recently, Wang et al. [20] showed that the majority of cadmium taken up by *Landoltia punctata* was associated with the cell wall, whereas the remaining pool could be found in the soluble fraction and in the organellar fraction. The authors further showed that over 80% of the protein-bound cadmium pool was associated with the albumin and globumin classes of proteins. However, the exact identity of the major cadmium-associating proteins or other cellular ligands that contributed to the cadmium accumulation in the duckweed remained unknown [20]. MTs serve as a major intracellular metal-chelating protein in plants, and the high expression of MT genes has been shown to correlate with metal tolerance and/or accumulation [21]. Thus, a fundamental understanding of the MT family in duckweeds may help to improve their capacity in metal bioremediation applications, as well as in the fortification of duckweeds to provide animal or human nutrition.

Owing to its complete genome sequence and genome simplicity, the greater duckweed *S. polyrhiza* was used in this study to examine the duckweed MT gene family. The first genome assembly was based on the *S. polyrhiza* clone 7498 [22]. The genome assembly of *S. polyrhiza* clone 7498 was updated [23,24], and another genome sequence of *S. polyrhiza,* clone 9509, was later released [25,26]. The genome sequences of other duckweeds, including *Lemna gibba*, *Lemna minor*, *Spirodela intermedia*, and *Wolffia australiana*, have also become available and should permit more comprehensive investigations of the duckweed MT gene family [27,28].

A search in the genome of *S. intermedia* and the other available duckweed species also failed to identify a homolog of Type 1 MTs (data not shown). This suggests that Type 1 MTs were lost from the genome of the ancestral duckweed species. It is possible that the function of root-specific MTs is not needed for duckweeds, although whether the expression of Type 1 MTs in the roots is replaced by that of the other remaining MT genes remains to be tested. If this is true, the absence of MT gene expression in duckweed roots may be attributable to a different mechanism of metal transport, distribution, or homeostasis in the root tissues compared to other non-aquatic plants.

Two homologs were found for the Type 2 MTs, although the predicted amino acid sequence of MT2b contained Cys residues primarily in the C-terminal half, suggesting that it is not a bona fide MT. It should also be noted that the predicted amino acid sequence from the coding sequence of Spipo0G0112500 described in the Phytozome database appeared to be shortened compared to the typical Type 2 MTs, and so we searched and found an alternative start codon at the position −810 nucleotide upstream from the originally predicted start codon, and we used this to derive the amino acid sequence that is homologous to other Type 2 MTs, as shown in this study. A homolog of a Type 3 MT and another of a Type 4 MT were identified in the genome of *S. polyrhiza*. In flowering plants, Type 3 MTs are typically expressed in ripening fruits, but the expression of Type 3 MTs can also be located in the leaves and in other tissues in plants, such as *A. thaliana*, which do not bear fleshy fruits [29]. The expression of Type 4 MTs is largely restricted to the seeds of flowering plants [30]. Although the function of seed-specific MTs is not yet clear, it is believed that they function in the storage of essential nutrients such as zinc and copper for the embryo and the early seedling during germination [31]. As with most duckweeds, *S. polyrhiza* propagates primarily through asexual vegetative budding. During an unfavorable growth period, *S. polyrhiza* can also form turions, which are a form of dormant vegetative tissues and resume growth after the harsh period concludes. On rare occasions, *S. polyrhiza* can flower and produce seeds [32]. The mRNA expression of *SpMT4* was not found in the RT-PCR analysis of whole plant tissues, and the hypothetical expression of *SpMT4* in the seeds remains to be investigated in the future.

The qRT-PCR analysis showed that the expression level of *SpMT2a* transcripts was upregulated in response to the cadmium treatment, and it was only slightly upregulated by the copper treatment, suggesting that the *S. polyrhiza* MT may play a specific role in Cd tolerance and/or accumulation. In *A. thaliana* [29,33] and several flowering plants [34,35,36], the mRNA abundance of MT genes is upregulated primarily by copper stress. Thus, the transcriptional response of *SpMT2a* suggests that cadmium may pose a greater threat than copper to duckweed in its aquatic habitat. Alternatively, there may exist another mechanism that helps duckweed cope with excessive copper. A similar observation was made in the moss *Physciomitrella patents* whose MT genes also responded primarily to cadmium treatment, but not to copper [37]. The down-regulation of *SpMT3* in response to the cadmium and copper treatments was similar to the down-regulation of barley MTs during cadmium, copper, and zinc exposure [38]. While the purpose of the down-regulation of an MT gene is not yet clear, several explanations have been proposed, including a role of MTs in the homeostasis of intracellular ROS, e.g., to allow the transient accumulation of ROS to trigger a signaling cascade [39,40]. The hypothesis that *SpMT3* is not directly involved in the chelation of excess metal ions was further supported the finding that the heterologous expression of *SpMT2a*, but not of *SpMT3*, was able to increase the copper and cadmium tolerance of the yeast ∆*cup1* mutant. Nevertheless, since the expression levels of the SpMT genes in the transgenic yeast strains were not analyzed, it should be re-emphasized that the inability of SpMT3 to confer copper or cadmium tolerance could be attributed to the low protein stability and/or expression level in the yeast cells; this possibility should be investigated in the future. The specific contributions of individual duckweed MTs should also be tested using more concrete evidence, such as characterization of the loss-of-function mutants to verify their function as well as to investigate their additional roles under diverse physiological conditions.

The insights on the forms and potential functions of duckweed MTs gained from this and from future studies may contribute to a more efficient use of duckweeds in environmental cleanup, nutrient fortification, and other innovative applications. For instance, the expression levels of different MTs may be investigated for their correlation with the metal bioaccumulation levels among duckweed varieties to obtain varieties that have higher nutritional value or increased ability to remove toxic pollutants from the environment. On the other hand, the duckweed varieties that have lower MT expression and that hypo-accumulate toxic metals may be selected when duckweeds are cultivated for livestock feed or human food. As shown this study, the ability of SpMTs to confer metal tolerance when heterologously expressed in *S. cerevisiae* was considerably lower than that of CUP1, suggesting that duckweed MTs do not chelate metal ions as efficiently. Thus, it is possible to explore more efficient metal-chelating MT isoforms from different duckweed species or varieties. The use of genetically engineered duckweeds that overexpress a transgenic MT gene may also be investigated in the future.

## 4. Materials and Methods

### 4.1. Plant Material and Culture Conditions

The *S. polyrhiza* clone 5638 was used in this study. The *S. polyrhiza* clone was originally isolated from a local pond in Raleigh, North Carolina, USA, and it has previously been described [17]. The plant cultures were maintained in a 125 mL Erlenmeyer flask containing 30 mL sterile Hoagland solution under continuous white fluorescent light at approximately 50 µmol photon m^−2^ s^−1^ in a temperature-controlled growth room at 23–25 °C.

### 4.2. Identification and Annotation of Putative MT Genes

The *S. polyrhiza* clone 7498 genome database in Phytozome version 13 (JGI) was first examined using a keyword search for “metallothionein”. To identify additional MT homologs, the *S. polyrhiza* genome databases in GenBank (NCBI) were examined using a TBLASTN search against amino acid sequences of the MT gene products from *O. sativa* and *A. thaliana*, as well as previously identified MT sequences from several monocot plants including *Zea mays* ZmMT1 (P30571), *Allium sativum* AsMT2 (AAV80430), *Posidonia oceanica* PoMT2 (CAF31414), *Wolffia arrhizal* WaMT2 (ADB85769), *Elaeis guineensis* EgMT3 (CAB52585), *Musa acuminate* MaMT3 (DN239297), *Triticum aestivum* TaMT4 (P30569.2), *Hordeum vulgare* HvMT4 (CAD88267.1), *Sorghum bicolor* SbMT4 (XP_002467575.1), and *Brachypodium distachyon* BdMT4 (XP_003572023.1). The putative MT-like sequences from *S. polyrhiza* were then aligned with the representative MT sequences obtained from the GenBank database using ClustalW (EMBL-EBI) and further adjusted manually using BioEdit 7.2 (MBIO-NCSU). The neighbor-joining tree was constructed using MEGA-X [41]. To find the chromosomal location of the genes, the coding sequences of the *S. polyrhiza* 7498 MT-like genes were used in a BLASTN search against *S. polyrhiza* 9509 in the GenBank (NCBI) database.

### 4.3. RT-PCR Analysis of S. polyrhiza MT Gene Expression

The *S*. *polyrhiza* clone WY001 plants, each containing 3–4 fronds, were cultured in sterile Hoagland solution for 3 days under continuous white fluorescent light. The whole plant tissues from five plants were transferred to a 1.5 mL microcentrifuge tube and frozen in liquid nitrogen before being ground with a plastic homogenizer pestle. For the analysis of the transcriptional responses to metal stress, CuSO_4_ or CdCl_2_ was added from 100 mM filter-sterile stock solutions to the indicated final concentration during the last 24 h. Triplicate cultures were included for each treatment. RNA extraction was performed using the TRIzol reagent (Invitrogen, Waltham, MA, USA) followed by using the TriRNA purification kit (Geneaid Biotech, New Taipei City, Taiwan ROC) according to the manufacturer’s protocol. The cDNA was synthesized in a 20 μL reaction containing 0.5 μg of DNase-treated total RNA using the ImProm-II reverse transcriptase (Promega, Madison, WI, USA) and an oligo-dT primer. The PCR amplification was performed using gene-specific primers (Table 2), Taq DNA polymerase (Vivantis Technologies, Selangor Darul Ehsan, Malaysia), and 1 µL of the first-strand cDNA mixture. The PCR condition was as follows: initial denaturation at 95 °C for 2 min, followed by the indicated number of cycles of 95 °C for 15 s, 56 °C for 15 s, and 72 °C for 30 s, and the final extension period at 72 °C for 2 min. The sizes of the PCR amplicons were verified using 1.5% agarose gel electrophoresis (Supplemental Figure S1). For the quantitative RT-PCR analysis, 1 μL of the first-strand cDNA mixture was amplified in an Applied Biosystems 7500 real-time PCR system (Thermo Fisher Scientific, Waltham, MA, USA) with a KAPA SYBR^®^ Fast ABI Prizm qPCR master mix (Kapa Biosystem, Potters Bar, UK) using the gene-specific primers. The expression levels were normalized to that of the *SpACT2* gene, which has been used as an internal standard in previous studies [36].

### 4.4. Heterologous Expression of SpMT2a and SpMT3

For the first-strand cDNA mixture, 1 µL, prepared as described above, was amplified using KOD high-fidelity DNA polymerase (Toyobo, Osaka, Japan) and the gene-specific primers that annealed to the 5′ and 3′ untranslated regions of *SpMT2a* and *SpMT3* (Table 2). The PCR condition was as follows: initial denaturation at 94 °C for 2 min, followed by 25 cycles of 94 °C for 15 s, 58 °C for 5 s, and 68 °C for 5 s, and the final extension period at 68 °C for 1 min. The PCR amplicons were purified using a PCR cleanup kit (Geneaid, New Taipei City, Taiwan ROC), incubated with Taq DNA polymerase (Vivantis Technologies, Selangor Darul Ehsan, Malaysia) to add a 3′-A overhang, and then ligated into the pGEMT-easy cloning vector (Promega, Madison, WI, USA). The sequence fidelity was confirmed by DNA sequencing. The full-length coding sequences were transferred to the p424-GPD expression vector [42] using the EcoRI and SpeI restriction sites between the *GPD* promoter and *CYC1* terminator. The construction of p424-GPD::*CUP1* was completed as previously described [37]. The wild type (DTY3) and ∆*cup1* (DTY4) strains of *S. cerevisiae* were kindly provided by Dr. Dennis J. Thiele of the Duke University School of Medicine, USA. All of the vectors including the p424-GPD empty vector were transformed into the yeast ∆*cup1* strain using a Frozen-EZ yeast transformation kit (Zymo Research, Irvine, CA, USA) according to the manufacturer’s protocol. The yeast transformants were selected on synthetic complete agar medium lacking tryptophan (SC-Trp) from Himedia Laboratories (Maharashtra, India).

For the spot dilution assay, overnight *S. cerevisiae* cultures were inoculated in 50 mL of SC-Trp broth medium at the initial OD_600_ of 0.2 and then shaken at 30 °C until the OD_600_ reached 0.8–1.0. The cultures were diluted to an OD_600_ of 0.2 and then serially diluted in fresh SC-Trp broth. From the 1, 1/10, 1/50, 1/100, and 1/500 diluted cultures, samples of 3 µL were spotted on SC-Trp 2% agar medium or the medium supplemented with CuSO_4_ or CdCl_2_ at the indicated concentrations. After incubation at 30 °C for 2–3 days, the plates were photographed.

## 5. Conclusions

We showed that the *S. polyrhiza* genome contains four MT-like genes: *SpMT2a*, *SpMT2b*, *SpMT3*, and *SpMT4*, encompassing three of the four Types of MTs in flowering plants. Even though the *SpMT2b* gene is expressed in vegetative tissues, its predicted amino acid sequence lacks nine of the fourteen conserved Cys residues found in other Type 2 MTs, and it is unlikely to function as a bona fide MT. The transcript of *SpMT4* was not detected in the vegetative whole-plant tissues, which was in agreement with the typical seed-specific localization of Type 4 MTs. The transcript abundance of *SpMT2a*, but not of *SpMT3*, was upregulated in response to cadmium stress. The heterologous expression of *SpMT2a*, but not of *SpMT3*, conferred copper and cadmium tolerance in the *S. cerevisiae* ∆*cup1* mutant. Therefore, among the four *S. polyrhiza* MT-like genes, *SpMT2a* is a strong candidate for playing an important role in the metal tolerance and accumulation of duckweed. This hypothetical function should be further investigated. The application of this knowledge may contribute to a more efficient use of duckweed in the environmental cleanup of metal pollutants in the future.

## Figures and Tables

**Table 1:** Annotation of *S. polyrhiza* MT-like genes.

Gene	Transcript	Location	No. of a.a.	No. of Cys
*MT2a*	Spipo0G0112500	Chr. 7	78	14
*MT2b*	Spipo6G0071500	Chr. 5	60	5
*MT3*	Spipo14G0028700	Chr. 9	65	10
*MT4*	Spipo0G0175800	Chr. 18	82	17

**Table 2:** Oligonucleotide primers used in the study.

Primers	Sequence (5′ → 3′)	Expected Amplicon Size (bp)	Annealing Temperature (°C)
Primers for RT-PCR Analysis			
SpMT2a-F	TGACGAGAAGATGACCACCA	148	56
SpMT2a-R	TCATTTGCAGGTGCAGGGAT		
SpMT2b-F	ATGTCTTGCACCAGCGGGAAA	134	56
SpMT2b-R	GCAGCGACCATCTCGAACGACTC		
SpMT3-F	ACCCAGTGCGTGAAGAAGGGCAA	162	56
SpMT3-R	TCAATGGCCGCAGGAGCAGGTGG		
SpMT4-F	GACGTAGGAGCGACGCGAGG	188	56
SpMT4-R	CCAACGCCAGAGGAAGCACC		
SpACT-F	GCGACATCAAGGAGAAGCTG	213	56
SpACT-R	AGTTGTAGGTGGTCTCGTGG		
Primers for heterologous expression			
SpMT2a-ORF-F	GAAGATGTCTTGCTGCGGAG	258	58
SpMT2a-ORF-R	CCTTCATACAGGAAGCGTCC		
SpMT3-ORF-F	CCATGTCGAGCTGCGGCAACT	203	58
SpMT3-ORF-R	CGCTCAATGGCCGCAGGA		

## Data Availability

The data presented in this study are available on request from the corresponding author.

## Supplementary Materials

The following supporting information can be downloaded at: https://www.mdpi.com/article/10.3390/plants12010125/s1, Supplemental Figure S1. Electrophoretic analysis of the RT-PCR amplicons: (a) *SpMT2a*; (b) *SpMT2b*; (c) *SpMT3*; and (d) *SpACT2*. Total cDNA was synthesized from a whole-plant RNA extract using an oligo-dT primer, followed by the PCR amplification using gene-specific primers with the cDNA mixture (lane 1) or the water negative control (lane N). The PCR amplicons were analyzed under 1.5% agarose gel electrophoresis in comparison to a standard size marker (lane M).

## References

[1] Nielson K.B., Atkin C.L., Winge D.R. (1985). Distinct metal-binding configurations in metallothionein. J. Biol. Chem..

[2] Krężel A., Maret W. (2021). The bioinorganic chemistry of mammalian metallothioneins. Chem. Rev..

[3] Cobbett C., Goldsbrough P. (2002). Phytochelatins and metallothioneins: Roles in heavy metal detoxification and homeostasis. Annu. Rev. Plant Biol..

[4] Guo W.J., Meetam M., Goldsbrough P.B. (2008). Examining the specific contributions of individual Arabidopsis metallothioneins to copper distribution and metal tolerance. Plant Physiol..

[5] Acosta K., Appenroth K.J., Borisjuk L., Edelman M., Heinig U., Jansen M.A.K., Oyama T., Pasaribu B., Schubert I., Sorrels S. (2021). Return of the Lemnaceae: Duckweed as a model plant system in the genomics and postgenomics era. Plant Cell.

[6] Vu G.T.H., Fourounjian P., Wang W., Cao X.H., Cao X.H., Fourounjian P., Wang W. (2020). Future prospects of duckweed research and applications. The Duckweed Genomes.

[7] Gupta C., Prakash D. (2013). Duckweed: An effective tool for phyto-remediation. Toxicol. Environ. Chem..

[8] Vardhan K.H., Kumar P.S., Panda R.C. (2019). A review on heavy metal pollution, toxicity and remedial measures: Current trends and future perspectives. J. Mol. Liq..

[9] Ali Z., Waheed H., Kazi A.G., Hayat A., Ahmad M., Ahmad P. (2016). Chapter 16—Duckweed: An efficient hyperaccumulator of heavy metals in water bodies. Plant Metal Interaction.

[10] Xu H., Yu C., Xia X., Li M., Li H., Wang Y., Wang S., Wang C., Ma Y., Zhou G. (2018). Comparative transcriptome analysis of duckweed (*Landoltia punctata*) in response to cadmium provides insights into molecular mechanisms underlying hyperaccumulation. Chemosphere.

[11] Chen Y., Zhao X., Li G., Kumar S., Sun Z., Li Y., Guo W., Yang J., Hou H. (2021). Genome-wide identification of the nramp gene family in *Spirodela polyrhiza* and expression analysis under cadmium Stress. Int. J. Mol. Sci..

[12] Chyan C.L., Lee T.T., Liu C.P., Yang Y.C., Tzen J.T., Chou W.M. (2005). Cloning and expression of a seed-specific metallothionein-like protein from sesame. Biosci. Biotechnol. Biochem..

[13] Ekperusi A.O., Sikoki F.D., Nwachukwu E.O. (2019). Application of common duckweed (*Lemna minor*) in phytoremediation of chemicals in the environment: State and future perspective. Chemosphere.

[14] Ceschin S., Crescenzi M., Iannelli M.A. (2020). Phytoremediation potential of the duckweeds *Lemna minuta* and *Lemna minor* to remove nutrients from treated waters. Environ. Sci. Pollut. Res..

[15] Liu Y., Xu H., Yu C., Zhou G. (2021). Multifaceted roles of duckweed in aquatic phytoremediation and bioproducts synthesis. GCB Bioenergy.

[16] Yoksan R., Boontanimitr A., Klompong N., Phothongsurakun T. (2022). Poly(lactic acid)/thermoplastic cassava starch blends filled with duckweed biomass. Int. J. Biol. Macromol..

[17] Liu Y., Sanguanphun T., Yuan W., Cheng J.J., Meetam M. (2017). The biological responses and metal phytoaccumulation of duckweed *Spirodela polyrhiza* to manganese and chromium. Environ. Sci. Pollut. Res..

[18] Hu D., Cheng M., Hu K., Zhang W., Yang Y., Xu Q. (2019). Evaluation of cobalt hyperaccumulation and tolerance potential of the duckweed (*Lemna minor* L.). Ecotoxicol. Environ. Saf..

[19] Chaudhary E., Sharma P. (2019). Chromium and cadmium removal from wastewater using duckweed—*Lemna gibba* L. and ultrastructural deformation due to metal toxicity. Int. J. Phytoremediation.

[20] Wang X., Zhang B., Wu D., Hu L., Huang T., Gao G., Huang S., Wu S. (2021). Chemical forms governing Cd tolerance and detoxification in duckweed (*Landoltia punctata*). Ecotoxicol. Environ. Saf..

[21] Murphy A., Taiz L. (1995). Comparison of metallothionein gene expression and nonprotein thiols in ten Arabidopsis ecotypes. Correlation with copper tolerance. Plant Physiol..

[22] Wang W., Haberer G., Gundlach H., Gläßer C., Nussbaumer T., Luo M.C., Lomsadze A., Borodovsky M., Kerstetter R.A., Shanklin J. (2014). The *Spirodela polyrhiza* genome reveals insights into its neotenous reduction fast growth and aquatic lifestyle. Nat. Commun..

[23] An D., Zhou Y., Li C., Xiao Q., Wang T., Zhang Y., Wu Y., Li Y., Chao D.Y., Messing J. (2019). Plant evolution and environmental adaptation unveiled by long-read whole-genome sequencing of Spirodela. Proc. Natl. Acad. Sci. USA.

[24] Harkess A., McLoughlin F., Bilkey N., Elliott K., Emenecker R., Mattoon E., Miller K., Czymmek K., Vierstra R.D., Meyers B.C. (2021). Improved *Spirodela polyrhiza* genome and proteomic analyses reveal a conserved chromosomal structure with high abundance of chloroplastic proteins favoring energy production. J. Exp. Bot..

[25] Michael T.P., Bryant D., Gutierrez R., Borisjuk N., Chu P., Zhang H., Xia J., Zhou J., Peng H., El Baidouri M. (2017). Comprehensive definition of genome features in *Spirodela polyrhiza* by high-depth physical mapping and short-read DNA sequencing strategies. Plant J..

[26] Hoang P.N.T., Michael T.P., Gilbert S., Chu P., Motley S.T., Appenroth K.J., Schubert I., Lam E. (2018). Generating a high-confidence reference genome map of the Greater Duckweed by integration of cytogenomic, optical mapping, and Oxford Nanopore technologies. Plant J..

[27] Park H., Park J.H., Lee Y., Woo D.U., Jeon H.H., Sung Y.W., Shim S., Kim S.H., Lee K.O., Kim J.-Y. (2021). Genome of the world’s smallest flowering plant, *Wolffia australiana*, helps explain its specialized physiology and unique morphology. Commun. Biol..

[28] An D., Li C., Zhou Y., Wu Y., Wang W. (2018). Genomes and transcriptomes of duckweeds. Front. Chem..

[29] Guo W.-J., Bundithya W., Goldsbrough P.B. (2003). Characterization of the Arabidopsis metallothionein gene family: Tissue-specific expression and induction during senescence and in response to copper. New Phytol..

[30] Kawashima I., Kennedy T.D., Chino M., Lane B.G. (1992). Wheat *Ec* metallothionein genes. Like mammalian Zn^2+^ metallothionein genes, wheat Zn^2+^ metallothionein genes are conspicuously expressed during embryogenesis. Eur. J. Biochem..

[31] Ren Y., Liu Y., Chen H., Li G., Zhang X., Zhao J. (2012). Type 4 metallothionein genes are involved in regulating Zn ion accumulation in late embryo and in controlling early seedling growth in Arabidopsis. Plant Cell Environ..

[32] Fourounjian P., Slovin J., Messing J. (2021). Flowering and seed production across the Lemnaceae. Int. J. Mol. Sci..

[33] Zhou J., Goldsbrough P.B. (1995). Structure, organization and expression of the metallothionein gene family in Arabidopsis. Mol. Gen. Genet.

[34] Xu X., Duan L., Yu J., Su C., Li J., Chen D., Zhang X., Song H., Pan Y. (2018). Characterization analysis and heavy metal-binding properties of CsMTL3 in *Escherichia coli*. FEBS Open Bio.

[35] Schiller M., Hegelund J.N., Pedas P., Kichey T., Laursen K.H., Husted S., Schjoerring J.K. (2014). Barley metallothioneins differ in ontogenetic pattern and response to metals. Plant Cell Environ..

[36] Hsieh H.M., Liu W.K., Huang P.C. (1995). A novel stress-inducible metallothionein-like gene from rice. Plant Mol. Biol..

[37] Pakdee O., Songnuan W., Panvisavas N., Pokethitiyook P., Yokthongwattana K., Meetam M. (2019). Functional characterization of metallothionein-like genes from *Physcomitrella patens*: Expression profiling, yeast heterologous expression, and disruption of *PpMT1.2a* gene. Planta.

[38] Hegelund J.N., Schiller M., Kichey T., Hansen T.H., Pedas P., Husted S., Schjoerring J.K. (2012). Barley metallothioneins: MT3 and MT4 are localized in the grain aleurone layer and show differential zinc binding. Plant Physiol..

[39] Wong H.L., Sakamoto T., Kawasaki T., Umemura K., Shimamoto K. (2004). Down-regulation of metallothionein, a reactive oxygen scavenger, by the small GTPase OsRac1 in rice. Plant Physiol..

[40] Xue T., Li X., Zhu W., Wu C., Yang G., Zheng C. (2009). Cotton metallothionein GhMT3a, a reactive oxygen species scavenger, increased tolerance against abiotic stress in transgenic tobacco and yeast. J. Exp. Bot..

[41] Kumar S., Stecher G., Li M., Knyaz C., Tamura K. (2018). MEGA X: Molecular evolutionary genetics analysis across computing platforms. Mol. Biol. Evol..

[42] Mumberg D., Muller R., Funk M. (1995). Yeast vectors for the controlled expression of heterologous proteins in different genetic backgrounds. Gene.

