# Supplementary material for: Examination of the Metallothionein Gene Family in Greater Duckweed Spirodela polyrhiza
Source: Plants (Basel). 2022 Dec 27;12(1):125. doi: 10.3390/plants12010125 (PMC9824710; doi:10.3390/plants12010125)
Supplement: Supplementary file 1 [file plants-12-00125-s001.zip › plants-1992732-supplementary.pdf]

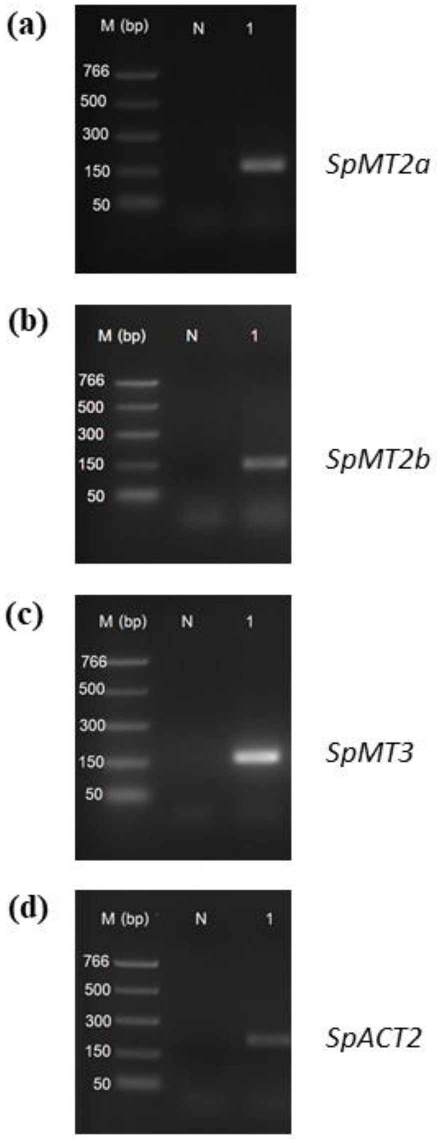

Figure S1. Electrophoretic analysis of the RT-PCR amplicons: (a) *SpMT2a*; (b) *SpMT2b*; (c) *SpMT3*; and (d) *SpACT2*. Total cDNA was synthesized from a whole-plant RNA extract using an oligo-dT primer, followed by the PCR amplification using gene-specific primers with the cDNA mixture (lane 1) or the water negative control (lane N). The PCR amplicons were analyzed under 1.5% agarose gel electrophoresis in comparison to a standard size marker (lane M).
